# On the Locally Polynomial Complexity of the Projection-Gradient Method for Solving Piecewise Quadratic Optimisation Problems

**DOI:** 10.3390/e23040465

**Published:** 2021-04-15

**Authors:** Agnieszka Prusińska, Krzysztof Szkatuła, Alexey Tret’yakov

**Affiliations:** 1 Faculty of Exact and Natural Sciences, Siedlce University, 08-110 Siedlce, Poland; Krzysztof.Szkatula@ibspan.waw.pl (K.S.); tret@uph.edu.pl (A.T.); 2 Systems Research Institute, Polish Academy of Sciences, 01-447 Warsaw, Poland; 3 Dorodnicyn Computing Centre of FRC CSC, Russian Academy of Sciences, 119333 Moscow, Russia

**Keywords:** optimisation problem, piecewise quadratic function, gradient method, system of linear inequalities, locally polynomial complexity

## Abstract

This paper proposes a method for solving optimisation problems involving piecewise quadratic functions. The method provides a solution in a finite number of iterations, and the computational complexity of the proposed method is locally polynomial of the problem dimension, i.e., if the initial point belongs to the sufficiently small neighbourhood of the solution set. Proposed method could be applied for solving large systems of linear inequalities.

## 1. Introduction

Let us consider the following optimisation problem:

(1) $$\min_{x\in R^{n}}{∥{\left(A\cdot x-b\right)}_{+}∥}^{2},$$

where $c_{+}:=\max\left\{c,0\right\}$, *A* is an m×n matrix, $A=\left\{a_{ij}\right\}$, $x\in R^{n}$, $x=\left\{x_{i}\right\}$, $b\in R^{m}$, $b=\left\{b_{j}\right\},$i=1,…,n, j=1,…m and ∥·∥ is the Euclidean norm of $R^{n}$.

In this paper, a method for solving the problem in (1) is proposed; moreover, the number of iterations (equivalent to the computational complexity) required by the proposed method with respect to *m* and *n* is locally polynomial, and in the worst-case scenario, it has a geometric convergence rate.

Let us define the set of solutions of (1) as follows

(2) $$X^{*}:=\left\{x^{*}\;\left|x^{*}=\arg\min_{x\in R^{n}}{∥{\left(A\cdot x-b\right)}_{+}∥}^{2}\right.\right\}.$$

If some point sufficiently close to the set $X^{*}$ of solutions to (1) is known, then it is possible to find a solution of (1) within a polynomial number of computational iterations; thus, the computational complexity is of the order of $O(m^{3}\cdot n^{3})$.

Many methods for solving (1) have been proposed (cf. Karmanov [1], Golikov and Evtushenko [2], Evtushenko and Golikov [3], Tretyakov [4], Tretyakov and Tyrtyshnikov [5] and Han [6]). All of these methods have reasonable computational complexity but, as mentioned above, to date, no strongly polynomial-time algorithm for solving (1) has been proposed. In studies by Tretyakov and Tyrtyshnikov [7] and Mangasarian [8], linear programming problems were solved by reducing them to the unconditional minimisation of strongly convex piecewise quadratic functions. A solution is obtained within a finite polynomial number of iterations if the starting point of the algorithm belongs to a sufficiently close neighbourhood of the unique solution to the problem. Unfortunately, the authors imposed severe limitations on the functions to be minimised: they should be strongly convex, the eigenvalues of the Hessian matrices should satisfy specific conditions, etc.

These results create significant limitations on the class of problems that can be solved: it is required that (1) has only one unique solution, etc. The solution method described by Tretyakov and Tyrtyshnikov [7] is based on exploiting information about the problem being solved by analysing a sufficiently small neighbourhood of an arbitrary solution of (1). Analogous methods were proposed by Facchinei et al. [9] for the forecasting (identification) of the active constraints in a sufficiently close neighbourhood of the solution to the problem. In papers by Tretyakov and Tyrtyshnikov [5] and Wright [10], locally polynomial methods for solving quadratic programming problems based on similar ideas were presented. Tretyakov [4] proposed the gradient projection method for solving (1); this method involves finding a solution of (1) in a finite number of iterations and is a combination of iterative and straightforward (e.g., Gaussian) methods.

This paper proposes a computational method for solving (1). When the starting point of the proposed method is sufficiently close to the set $X^{*}$ of solutions to (1), then its computational complexity is locally polynomial, i.e., it is of the order of $O(m^{3}\cdot n^{3})$.

We point out that solving a system of linear inequalities involves

(3) $$A\cdot x-b\leq 0_{m},$$

where the $0_{m}$ – *m*-dimensional vectors of zeroes can be reduced to solve the problem (1). This means that the number of computations required for establishing a solution (if a given system of linear inequalities has one) is locally polynomial.

Let us denote

(4) $$X:=\left\{x\in R^{n}\left|\;A\cdot x-b\leq 0_{m}\right.\right\}.$$

It is obvious that the set *X* might be empty in general, but our method, presented in this paper, either determines this situation in a locally polynomial number of computations or provides a solution to the system (3). The proposed method could be applied when solving large systems of linear inequalities, which appear in many practical, industrial applications, e.g., the simplex method (Pan [11]), Karmarkar’s method (Wright, [12]), Chubanov’s method (Roos [13]), and Fourier–Motzkin elimination method (Khachiyan [14], I. Šimeček, R. Fritsch, D. Langr, R. Lórencz [15]).

## 2. Definitions and Theoretical Results

Let

(5) $$\varphi \left(x\right)=\frac{1}{2}\cdot {∥{\left(A\cdot x-b\right)}_{+}∥}^{2}.$$

**Theorem** **1.** *The function φ(x) is convex and has a nonempty set of minimal values*

(6) $$X^{*}:=\left\{x^{*}\in R^{n}\;\left|\;\varphi \left(x^{*}\right)=\min_{x\in R^{n}}\varphi \left(x\right)\right.\right\}.$$

**Proof.**  Theorem 1 follows immediately from the well-known features of quadratic-type convex functions (see, e.g., [16]). □

It is obvious that the elements $x^{*}\in X^{*}$, cf. (6) satisfy

(7) $$\varphi^{\prime }{\left(x^{*}\right)}^{⊤}=\sum_{i=1}^{m}{\left(\langle a_{i},x^{*}\rangle -b_{i}\right)}_{+}\cdot a_{i}=0_{n}=A^{T}\cdot {\left(A\cdot x^{*}-b\right)}_{+},$$

where $a_{i}^{T}$ is the *i*th row of matrix *A*.

Therefore, in the general case, our goal is to solve the following equation

(8) $$\varphi^{\prime }{\left(x\right)}^{⊤}=\sum_{i=1}^{m}{\left(\langle a_{i},x\rangle -b_{i}\right)}_{+}\cdot a_{i}=A^{T}\cdot {\left(A\cdot x-b\right)}_{+}=0_{n},\;\mathrm{where}\;x\in R^{n}.$$

In the sequel, $x^{*}$ stands for an arbitrary element of $X^{*}$ (the minimum point of φ). If the minimum value of φ is equal to zero, then $X=X^{*}$, and if the minimum value of φ is positive, then X=∅. Let us denote

$$f_{i}\left(x\right):=\langle a_{i},x\rangle -b_{i},\;i\in D=\left\{1,\ldots m\right\},$$

and

(9) $$\begin{array}{cc} J_{0}\left(x\right) & :=\left\{i\in D\;\left|\;f_{i}\left(x\right)=0\right.\right\},\;J_{-}\left(x\right):=\left\{i\in D\;\left|\;f_{i}\left(x\right)<0\right.\right\},\\ J_{+}\left(x\right) & :=\left\{i\in D\;\left|\;f_{i}\left(x\right)>0\right.\right\}, \end{array}$$

where $f_{i}\left(x\right)$ is introduced to simplify the definitions of the sets $J_{0}\left(x\right)$ and $J_{+}\left(x\right)$.

According to (7) and the above notations, $x^{*}$ should satisfy the formula

(10) $$\sum_{i\in J_{0}\left(x^{*}\right)\cup J_{+}\left(x^{*}\right)}{\left(\langle a_{i},x^{*}\rangle -b_{i}\right)}_{+}\cdot a_{i}=0_{n}.$$

The formula (10) is equivalent to a condition that should be satisfied at point $x^{*}$

(11) $$\begin{array}{c} \sum_{i\in J_{0}\left(x^{*}\right)\cup J_{+}\left(x^{*}\right)}\left(\langle a_{i},x^{*}\rangle -b_{i}\right)\cdot a_{i}=0_{n}, \end{array}$$

In (11), it is considered that

$${\left(\langle a_{i},x^{*}\rangle -b_{i}\right)}_{+}=\langle a_{i},x^{*}\rangle -b_{i},\;i\in J_{0}\left(x^{*}\right)\cup J_{+}\left(x^{*}\right).$$

This, in turn, means that, in the general case, we should solve the following equations

(12) $$\sum_{i\in J_{0}\left(x\right)\cup J_{+}\left(x\right)}{\left(\langle a_{i},x\rangle -b_{i}\right)}_{+}\cdot a_{i}=0_{n},$$

or

(13) $$\begin{array}{cc} \sum_{i\in J_{+}\left(x\right)} & {\left(\langle a_{i},x\rangle -b_{i}\right)}_{+}\cdot a_{i}=0_{n},\\ \; & \langle a_{i},x\rangle -b_{i}=0,\;i\in J_{0}\left(x\right). \end{array}$$

Without loss of generality, we may denote

$$J_{-}\left(x^{*}\right):=\left\{1,\ldots ,l\right\},\;J_{0}\left(x^{*}\right):=\left\{l+1,\ldots ,p\right\},\;J_{+}\left(x^{*}\right):=\left\{p+1,\ldots ,m\right\},\;$$

where l≤p≤m.

The main idea exploited in this paper is based on the following Lemma. For ε>0 we set $U_{\varepsilon }\left(x^{*}\right):=\left\{x\in R^{n}\;:\;∥x-x^{*}∥\leq \varepsilon \right\}$.

**Lemma** **1.** Let $x^{*}$ be a solution to the problem (1). Then, there exists ε>0, such that for any $x\in U_{\varepsilon }\left(x^{*}\right)$, the inequality $f_{i}\left(x\right)\geq 0$ implies the inequality $f_{i}\left(x^{*}\right)\geq 0$.

**Proof.**  If $i\in J_{-}\left(x^{*}\right),$ that is $f_{i}\left(x^{*}\right)<0$, then, by continuity of the function $f_{i}$, there exists $\varepsilon_{i}>0$, such that $f_{i}\left(x\right)<0$ for all $x\in U_{\varepsilon_{i}}\left(x^{*}\right)$. Set

$$\varepsilon =\min_{i\in J_{-}\left(x^{*}\right)}\varepsilon_{i}.$$

Then, for all $i\in J_{-}\left(x^{*}\right)$ and for all $x\in U_{\varepsilon }\left(x^{*}\right)$, we have $f_{i}\left(x\right)<0$. Consequently, if there exists $x\in U_{\varepsilon }\left(x^{*}\right)$ such that $f_{i}\left(x\right)\geq 0$ with some i∈{1,…,m}, then $i\notin J_{-}\left(x^{*}\right)$, that is $f_{i}\left(x^{*}\right)\geq 0$. □

By virtue of the above lemma, in a sufficiently small neighbourhood of some fixed point $x^{*}\in X^{*}$, for every $\bar{x}\in U_{\varepsilon }\left(x^{*}\right)$, the following hold

$$J_{0}\left(\bar{x}\right)\subseteq J_{0}\left(x^{*}\right)\;\mathrm{and}\;J_{+}\left(\bar{x}\right)\subseteq J_{0}\left(x^{*}\right)\cup J_{+}\left(x^{*}\right),\;J_{-}\left(\bar{x}\right)\subseteq J_{0}\left(x^{*}\right)\cup J_{-}\left(x^{*}\right).$$

Now, our goal is to correctly define the sets $J_{0}\left(x^{*}\right)$ and $J_{+}\left(x^{*}\right)$ based on the information gained at point $\bar{x}\in U_{\varepsilon }\left(x^{*}\right)$. Let us denote

$${\bar{J}}_{0}\left(\bar{x}\right):=J_{0}\left(\bar{x}\right),\;{\bar{J}}_{+}\left(\bar{x}\right):=J_{+}\left(\bar{x}\right),\;{\bar{J}}_{-}\left(\bar{x}\right):=J_{-}\left(\bar{x}\right).$$

Let $A(\bar{x})$ and $b(\bar{x})$ represent the matrix and vector obtained from *A* and *b*, respectively. The rows of $A(\bar{x})$ and the coefficients of $b(\bar{x})$ correspond to the index set, which is defined by ${\bar{J}}_{0}\left(\bar{x}\right)\cup {\bar{J}}_{+}\left(\bar{x}\right)$. In this case, Equations (12)–(13) may be rewritten as

(14) $$\begin{array}{cc} A^{T}\left(\bar{x}\right) & \cdot \left(A\left(\bar{x}\right)\cdot x-b\left(\bar{x}\right)\right)=0_{n},\\ \langle a_{i},x\rangle & -b_{i}=0,\;i\in {\bar{J}}_{0}\left(\bar{x}\right). \end{array}$$

Let $\bar{A}\left(\bar{x}\right)$ denote the matrix in the equations in (14) corresponding to the maximum set of linearly independent rows, and let $\bar{b}\left(\bar{x}\right)$ denote the corresponding vector of constant terms in (14).

The equations in (14) may be reformulated in the following way

(15) $$\bar{A}\left(\bar{x}\right)\cdot x-\bar{b}\left(\bar{x}\right)=0_{n}.$$

Let us observe that, at point $x^{*}$, the following holds

(16) $$A^{T}\left(x^{*}\right)\cdot {\left(A\left(x^{*}\right)\cdot x^{*}-b\left(x^{*}\right)\right)}_{+}=0_{n}.$$

This, in turn, means that

(17) $$\bar{A}\left(x^{*}\right)\cdot x^{*}-\bar{b}\left(x^{*}\right)=0_{n}.$$

(18) $$\begin{array}{cc} M\left(\bar{x}\right):=\{x\in R^{n}| & \sum_{i\in {\bar{J}}_{0}\left(\bar{x}\right)\cup {\bar{J}}_{+}\left(\bar{x}\right)}\left(\langle a_{i},x\rangle -b_{i}\right)\cdot a_{i}=0_{n}\\ \; & \left.\;\mathrm{and}\;\langle a_{j},x\rangle -b_{j}=0,\;\mathrm{where}\;j\in {\bar{J}}_{0}\left(\bar{x}\right)\right\}. \end{array}$$

If the rank of a matrix *B* of size r×n is equal to *r*, then the pseudoinverse matrix (operator) $B^{+}$ may be defined as $B^{+}:=B^{T}\cdot {\left(B\cdot B^{T}\right)}^{-1}$. We denote the quadratic matrix n×n orthogonally projected on the space containing the rows of matrix *B* as ${\left(B^{T}\right)}^{\mathrm{II}}:=B^{T}{\left(B\cdot B^{T}\right)}^{-1}\cdot B=B^{+}\cdot B$, and its projection on the orthogonal complement of the matrix is denoted as ${\left(B^{T}\right)}^{⊥}:=$$I-{\left(B^{T}\right)}^{\mathrm{II}}$, where *I* is an all-ones matrix of size n×n.

Let a point $z(\bar{x})$ be the projection of point $\bar{x}$ on the set $M(\bar{x})$. Let us observe that $x^{*}\in M\left(\bar{x}\right)$ if $\bar{x}\in U_{\varepsilon }\left(x^{*}\right)$ and ε is sufficiently small.

Moreover, if the constraints at point $z(\bar{x})$ are $f_{i}\left(z\left(\bar{x}\right)\right)\leq 0$ for a certain $i\in {\bar{J}}_{+}\left(\bar{x}\right)$, then we define the set $I_{-}$ in the following way

$$I_{-}=\left\{i\in {\bar{J}}_{+}\left(\bar{x}\right)|f_{i}\left(z\left(\bar{x}\right)\right)\leq 0\right\};\;I_{-}\subseteq J_{0}\left(x^{*}\right).$$

Otherwise, if the constraints at point $z(\bar{x})$ are $f_{i}\left(z\left(\bar{x}\right)\right)\geq 0$ for a certain $i\in {\bar{J}}_{-}\left(\bar{x}\right)$, we define the set $I_{+}$ in an analogous way

$$I_{+}=\left\{i\in {\bar{J}}_{-}\left(\bar{x}\right)|f_{i}\left(z\left(\bar{x}\right)\right)\geq 0\right\};\;\;I_{+}\subseteq J_{0}\left(x^{*}\right).$$

Now, we redefine ${\bar{J}}_{0}\left(\bar{x}\right)$, ${\bar{J}}_{+}\left(\bar{x}\right)$ and ${\bar{J}}_{-}\left(\bar{x}\right)$ as follows

(19) $${\bar{J}}_{0}\left(\bar{x}\right):={\bar{J}}_{0}\left(\bar{x}\right)\cup I_{-}\cup I_{+},\;\;{\bar{J}}_{+}\left(\bar{x}\right):={\bar{J}}_{+}\left(\bar{x}\right)\backslash I_{-},\;\;{\bar{J}}_{-}\left(\bar{x}\right):={\bar{J}}_{-}\left(\bar{x}\right)\backslash I_{+}.$$

Next, we project point $\bar{x}$ on the new set $M(\bar{x})$, cf. (18), and a new point $z(\bar{x})$ is obtained.

Let

(20) $$z\left(x\right)=P_{M(\bar{x})}\left(x\right)={\left(A^{T}\left(\bar{x}\right)\right)}^{⊥}\cdot x+{\bar{A}}^{+}\left(\bar{x}\right)\cdot \bar{b}\left(\bar{x}\right)$$

define the operator for the projection of point *x* on set $M(\bar{x}).$

## 3. Algorithm for Finding the Solution of (1)

In this section, the algorithm designed to find the solution to (1) is presented. The main idea of this algorithm is based on information related to a current point $\bar{x}$ belonging to a sufficiently small neighbourhood of the point $x^{*}\in X^{*}$. We also demonstrate how to find such a point. The proposed method comprises two algorithms. The starting point of the method can be arbitrary, because Algorithm 2 (gradient method with a special step selection) starts at an arbitrary point and, on a certain iteration, it will provide a point arbitrarily close to the solution set. Therefore, Algorithm 1 could start at the point specified by Algorithm 2.

**Algorithm** **1.** ***Initialisation Step**: For the current point $\bar{x}$, the sets of indices $J_{0}\left(\bar{x}\right)$, $J_{-}\left(\bar{x}\right)$ and $J_{+}\left(\bar{x}\right)$ are defined according to (9). If set $J_{+}\left(\bar{x}\right)=\emptyset$, then $\bar{x}$ is the solution of (1) and Algorithm 1 is terminated. Otherwise, the Main Recursive Step is performed.*

***Main Recursive Step**: Let* $z(\bar{x})$ *, the projection of point* $\bar{x}$ *on the set* $M(\bar{x})$ *, be defined according to (20). We check if the following condition is satisfied*

(21) $$I_{+}=\emptyset \;\;\mathrm{and}\;\;I_{-}=\emptyset .$$

**Checking Step**: If (21) holds, then $z\left(\bar{x}\right)\in X^{*}$, and Equation (10) is satisfied; $z(\bar{x})$ is the solution of (1), as defined in (2), and Algorithm 1 is terminated. Otherwise, if for certain values of i∈D the condition (21) is violated and $i\in I_{+}\cup I_{-}$, we define ${\bar{J}}_{0}\left(\bar{x}\right)$, ${\bar{J}}_{+}\left(\bar{x}\right)$ and ${\bar{J}}_{-}\left(\bar{x}\right)$ according to (19), $M(\bar{x})$ is redefined according to (18), and the Main Recursive Step is repeated.

The set D is finite, and $\left|D\right|=m$; therefore, the number of changes to the index sets ${\bar{J}}_{0}\left(\bar{x}\right)$, ${\bar{J}}_{+}\left(\bar{x}\right)$ and ${\bar{J}}_{-}\left(\bar{x}\right)$ does not exceed *m*, and finally, the point $z(\bar{x})$ fulfilling (12) is established. This means that $z(\bar{x})$ is the solution of (1), as defined in (2).

It is of utmost importance that $\bar{x}$ belongs to a sufficiently small neighbourhood of the point $x^{*}$, because otherwise, $z(\bar{x})$ may not satisfy (12). If this is not the case, it is necessary to find another point $\bar{x}$ that is closer to $x^{*}$. The process for accomplishing this is described below.

**Theorem** **2.** For a sufficiently small ε>0 and for every $\bar{x}\in U_{\varepsilon }\left(x^{*}\right)$, Algorithm 1 provides $z^{*}=z\left(\bar{x}\right)$ as the solution for

(22) $$\varphi^{\prime }\left(x\right)=A^{T}\left(x\right)\cdot {\left(A(x)\cdot x-b\right)}_{+}=0_{n},$$

 *and this is equivalent to finding the solution for (12) within a number of iterations of the order of $0(m^{3}\cdot n^{3})$.*

**Proof.**  The proof is based on the observation that for $\bar{x}$ belonging to a sufficiently small neighbourhood of the point $x^{*}$, according to Lemma 1, the constraints $f_{i}\left(\bar{x}\right)\geq 0$ correspond to constraints $f_{i}\left(x^{*}\right)\geq 0$. Therefore,

$${\bar{J}}_{0}\left(\bar{x}\right)\cup {\bar{J}}_{+}\left(\bar{x}\right)\subseteq J_{0}\left(x^{*}\right)\cup J_{+}\left(x^{*}\right).$$

Let us determine $z(\bar{x})$ as the projection of the point $\bar{x}$ on the set $M(\bar{x})$, which is defined according to (18). It may happen that the set ${\bar{J}}_{0}\left(\bar{x}\right)$ becomes enlarged. However, the number of iterations required when ${\bar{J}}_{0}\left(\bar{x}\right)$ becomes enlarged does not exceed *m*, the number of elements in the set *D*. Therefore, at some iteration, (21) is satisfied. This means that $z(\bar{x})$ satisfies (12) or, equivalently, $\varphi^{\prime }\left(z\left(\bar{x}\right)\right)=0_{n}$. This demonstrates that $z(\bar{x})$ is the solution for (1), as defined in (2). The computational complexity of establishing each projection $z(\bar{x})$ is of order $0(m^{2}\cdot n^{3})$; this process takes the computational effort related to the multiplications of matrices into account. The number of iterations does not exceed *m* and, therefore, the overall computational complexity is of order $0(m^{3}\cdot n^{3})$. □

To complement the presentation of this chapter, the gradient method for establishing $\bar{x}$ belonging to the sufficiently small neighbourhood $U_{\varepsilon }\left(x^{*}\right)$ of some fixed solution $x^{*}\in X^{*}$ to (1) is described. This gradient method has the following scheme

(23) $$x_{k+1}=x_{k}-\alpha \cdot \varphi^{\prime }\left(x_{k}\right)$$

where $\alpha =\frac{1}{L}$ and gradient $\varphi^{\prime }\left(x_{k}\right)$ fulfils the Lipschitz condition

$$\left|\varphi^{\prime }\left(x_{k+1}\right)-\varphi^{\prime }\left(x_{k}\right)\right|\leq L\cdot \left|x_{k+1}-x_{k}\right|\;\mathrm{where}\;L=2\cdot ∥A^{T}\cdot A∥.$$

The convergence of the gradient method (23) is considered in the following theorem, cf. Karmanov [1].

**Theorem** **3.** *Let $x_{0}\in R^{n}$ and the sequence $\left\{x_{k}\right\}$, k=0,1,2…, be constructed according to (23). Then,*

$$x_{k}\to x^{*},\;x^{*}\in X^{*},\;\mathrm{where}\;k\to \infty \;\mathrm{and}\;∥x_{k+1}-y∥<∥x_{k}-y∥\;\forall \;y\in X^{*}.$$

**Proof.**  The scheme in (23) produces a sequence that converges to a certain $x^{*}\in X^{*}$. Moreover, for every sufficiently small ε>0, there exists $\bar{k}=k\left(\varepsilon \right)$ such that $\left\{x_{k}\right\}\in U_{\varepsilon }\left(x^{*}\right)$, for all $k\geq \bar{k}$. This, in turn, means that at iteration $\bar{k}$, the hypothesis of Theorem 2 is satisfied, and we obtain a solution to (1). □

Now, we have all the necessary prerequisites to present the solution algorithm for (3).

**Algorithm** **2.** ***Initialisation Step**: Let k=0, and let $x_{0}\;$ be an arbitrary point in $R^{n}$.*

***Main Recursive Step**: Let*

$$x_{k+1}=x_{k}-\alpha \cdot \varphi^{\prime }\left(x_{k}\right).$$

***Checking Step**: If $z(x_{k})$ is the solution for (3), then Algorithm 2 is terminated. Otherwise, we set k:=k+1, and the Main Recursive Step is repeated.*

**Theorem** **4.** There exists a finite $\bar{k}$ such that $z\left(x_{\bar{k}}\right)\in X^{*}$ and $z(x_{\bar{k}})$ is the solution for (3).

**Proof.**  The sequence $\left\{x_{k}\right\}$ converges to a fixed $x^{*}\in X^{*}$ and therefore, at a certain iteration $\bar{k}$, the hypothesis of Theorem 2 is satisfied, and we obtain the solution $z^{*}=P_{M(x_{\bar{k}})}\in X^{*}$. □

Theorem 4 allows us to establish whether (3) has a solution or not.

**Corollary** **1.** *If*

$$z^{*}\in X,$$

then $z^{*}$ is the solution of (3). Otherwise (3) has no solutions.

## 4. Conclusions and Appendix

As previously mentioned, the locally polynomial complexity estimate is valid only if the starting point of the proposed method belongs to a sufficiently small neighbourhood of the set of solutions $X^{*}$. To reach such a desired point, the gradient method (23) is used. There are accelerated gradient methods (see those of Nesterov [17] and Poliak [18]), but these methods do not guarantee monotonic convergence to a set of solutions $X^{*}$. The method presented in this paper monotonically converges to a certain point $x^{*}$, $x^{*}\in X^{*}$. It is obvious that the point $x^{*}$ depends on the initial point $x_{0}$ and, therefore, the number of iterations required by the gradient method for entering the proper neighbourhood of point $x^{*}$ depends on the position of the initial point $x_{0}$. Moreover, the ε radius of the neighbourhood of point $x^{*}$, which the gradient method should reach, is unknown in the general case and depends on the specific problem being considered. However, it appears that we can guarantee a geometric convergence rate for the gradient method (23) while minimising piecewise quadratic functions of the form (5).

Namely, for every strongly convex function ψ(x), the gradient method (23) has a geometric convergence rate, i.e.,

$$\psi \left(x_{k}\right)-\psi^{*}\leq c\cdot \delta^{k},\;\mathrm{where}\;0<\delta <1,\;c>0,$$

where *c* is a constant that is independent of the size of the problem but depends on the initial point $x_{0}$. In the general case, for functions that are not convex in the strongest sense, there is no proof of the geometric convergence of the gradient method (23). However, in the case where the function φ(x) is given by (5), it is possible to prove the geometric convergence of the gradient method (23). Let

$$\begin{array}{cc} l(x_{k}) & =\left\{x^{*}+\beta \cdot \left(x_{k}-x^{*}\right),\;\beta \geq 0\right\}\;\mathrm{and}\;M\left(s_{k}\right)=\left\{x^{*}+\beta \cdot s_{k},\;\beta \geq 0\right\},\;\\ s_{k} & =\frac{x_{k}-x^{*}}{∥x_{k}-x^{*}∥}. \end{array}$$

The theorem presented below proves the strong convexity of the function φ(x) in the cone of convergence.

**Theorem** **5.** *The elements of the sequence $\left\{x_{k}\right\},$ defined by (23), belong to the cone of strong convexity of the function φ(x), namely ∀x,$\;y\in l(x_{k})$, the function φ(x) is uniformly strongly convex for the sequence $\left\{x_{k}\right\}$, i.e.,*

(24) $$\varphi \left(\lambda \cdot x+\left(1-\lambda \right)\cdot y\right)\leq \lambda \cdot \varphi \left(x\right)+\left(1-\lambda \right)\cdot \varphi \left(y\right)-\gamma \cdot \lambda \cdot \left(1-\lambda \right)\cdot {∥x-y∥}^{2}$$

*where $\lambda \in \left[0,1\right]$, $x,y\in l(x_{k})$, k=0,1…, and γ>0.*

**Proof.**  First, it should be pointed out that because the second derivative of the function φ(x) has a finite number of points of discontinuity $\bar{S}\in R^{n}$ in every direction, i.e., on the ray $x^{*}+\lambda \cdot \bar{S}$, there exists σ>0 such that on the closed interval $\left[x^{*},x^{*}+\sigma \cdot \bar{S}\right]$, the function φ(x) has a continuous second derivative that obviously depends on $\bar{S}$. Let us assume that the theorem does not hold, i.e., there is not γ>0, such that (24) holds. This means that for

$$l\left(x_{k}\right)=\left\{x^{*}+\beta \cdot s_{k},\;\beta \geq 0\right\}$$

the following must hold

(25) $$\frac{\partial^{2}\varphi \left(x^{*}\right)}{\partial s_{k}^{2}}=\gamma_{k}\to 0\;\mathrm{when}\;k\to \infty ,$$

or

$$\frac{\partial^{2}\varphi \left(x^{*}\right)}{\partial s_{k}^{2}}=\langle A^{T}\cdot A\cdot s_{k},s_{k}\rangle =\gamma_{k}\to 0\;\mathrm{when}\;k\to \infty .$$

For vector $s=\lim_{k\to \infty }s_{k}$, the following condition $\langle A^{T}\cdot A\cdot s,s\rangle =0$ holds, or, due to the construction of φ(x),

$$\varphi \left(x^{*}+\beta \cdot s\right)=0=\varphi \left(x^{*}\right)=\min{∥{\left(A\cdot x-b\right)}_{+}∥}^{2},$$

where $\beta \in [0,\bar{\beta }]$, $\bar{\beta }>0$ is a certain fixed constant. Let $x_{k}^{*}$ be (locally) the projection of $x_{k}$ on the set $M\left(s\right)\in X^{*}$. Then, due to $s_{k}\to s$, k→∞, we have

(26) $$∥x_{k}-x_{k}^{*}∥=\delta_{k}\cdot ∥x_{k}-x^{*}∥,\;\mathrm{where}\;\delta_{k}\to 0,\;k\to \infty .$$

Let us set $\delta_{k}$ sufficiently small and consider the points $x_{k+r}$, r=1,2,…. Then, according to Theorem 3, we have

(27) $$∥x_{k+r}-x_{k}^{*}∥<∥x_{k}-x_{k}^{*}∥.$$

On the other hand, according to (26), when r→∞

$$\begin{array}{cc} ∥x_{k+r}-x_{k}^{*}∥ & \geq ∥x_{k}^{*}-x^{*}∥-∥x_{k+r}-x^{*}∥\geq \\ ∥x_{k}-x^{*}∥ & -∥x_{k}-x_{k}^{*}∥-∥x_{k+r}-x^{*}∥\geq \\ \frac{1}{\delta_{k}}∥x_{k}-x_{k}^{*}∥ & -∥x_{k}-x_{k}^{*}∥-∥x_{k+r}-x^{*}∥>∥x_{k}-x_{k}^{*}∥. \end{array}$$

This is contradictory to (27), and therefore Theorem 5 holds. □

Theorem 5 allows for the estimation of the convergence rate of the gradient method (23).

**Theorem** **6.** *Under the assumptions of Theorem 5 for the sequence $\left\{x_{k}\right\},$ constructed according to (23), the following convergence rates hold*

(28) $$\varphi \left(x_{k}\right)-\varphi^{*}\leq c_{1}\cdot \tau^{k}\;\mathrm{and}\;∥x_{k}-x^{*}∥\leq c_{2}\cdot \tau^{\frac{k}{2}},$$

*where τ∈(0,1), and $c_{1}$ and $c_{2}$>0; the constants $c_{1}$, $c_{2}$ are independent of the value of k but depend on the initial point $x_{0}$.*

**Proof.**  Let us denote

$$\mu_{k}=\varphi \left(x_{k}\right)-\varphi^{*}.$$

For the sequence $\left\{x_{k}\right\}$ and $q\in \left(\frac{1}{2},1\right)$ the following holds

(29) $$\begin{array}{cc} \varphi \left(x_{k}\right)-\varphi \left(x_{k+1}\right) & \geq \alpha \cdot q\cdot {∥\varphi^{\prime }\left(x_{k}\right)∥}^{2}\geq \alpha \cdot q\cdot {\langle \varphi^{\prime }\left(x_{k}\right),s_{k}\rangle }^{2}=\frac{\partial^{2}\varphi \left(x_{k}\right)}{\partial s_{k}^{2}}\geq \\ \; & \geq \alpha \cdot q\cdot \gamma^{2}\cdot \left(\varphi \left(x_{k}\right)-\varphi^{*}\right) \end{array}$$

or, equivalently,

$$\mu_{k}-\mu_{k+1}\geq \alpha \cdot q\cdot \gamma^{2}\mu_{k}.$$

Therefore, for τ∈(0,1), the following holds

$$\mu_{k}\leq c_{1}\cdot \tau^{k}\;\mathrm{or},\;\mathrm{equivalently},\;\varphi \left(x_{k}\right)-\varphi^{*}\leq c_{1}\cdot \tau^{k}.$$

This proves the first part of (28), while the latter part of (28) follows from the strong convexity of the function φ(x) in the cone of convergence. □

The conduction computational experiments and comparison of the presented method with other methods in the literature remains a topic for future research.
